# SUMOylation patterns and signature characterize the tumor microenvironment and predict prognosis in lung adenocarcinoma

**DOI:** 10.3389/fcell.2023.1094588

**Published:** 2023-04-13

**Authors:** Zhike Chen, Jian Yang, Lijuan Tang, Xue Sun, Yu Li, Ziqing Sheng, Hao Ding, Chun Xu, Xin Tong, Jun Zhao

**Affiliations:** ^1^ Institute of Thoracic Surgery, The First Affiliated Hospital of Soochow University, Suzhou, China; ^2^ Department of Thoracic Surgery, The First Affiliated Hospital of Soochow University, Suzhou, China; ^3^ Dalian Medical University, Dalian, China; ^4^ Department of Pathology, Affiliated Hospital of Nantong University, Nantong, China; ^5^ Department of Pathology, Zhujiang Hospital, Southern Medical University, Guangzhou, China

**Keywords:** SUMOylation, tumor microenvironment, immune therapy, prognosis, lung adenocarcinoma

## Abstract

**Background:** Recent studies have revealed that SUMOylation modifications are involved in various biological processes, including cancer development and progression. However, the precise role of SUMOylation in lung adenocarcinoma (LUAD), especially in the tumor immune microenvironment, is not yet clear.

**Methods:** We identified SUMOylation patterns by unsupervised consensus clustering based on the expression of SUMOylation regulatory genes. The tumor microenvironment in lung adenocarcinoma was analyzed using algorithms such as GSVA and ssGSEA. Key genes of SUMOylation patterns were screened for developing a SUMOylation scoring model to assess immunotherapy and chemotherapy responses in lung adenocarcinoma patients. Experiments were conducted to validate the differential expression of model genes in lung adenocarcinoma. Finally, we constructed a nomogram based on the SUMOylation score to assess the prognosis of individual lung adenocarcinoma patients.

**Results:** Two patterns of SUMOylation were identified, namely, SUMO-C1, which showed anti-tumor immune phenotype, and SUMO-C2, which showed immunosuppressive phenotype. Different genomic subtypes were also identified; subtype gene-T1 exhibited a reciprocal restriction between the immune microenvironment and stromal microenvironment. High SUMOylation scores were indicative of poor lung adenocarcinoma prognosis. SUMOylation score was remarkably negatively correlated with the infiltration of anti-tumor immune cells, and significantly positively correlated with immune cells promoting immune escape and immune suppression. In addition, patients with low scores responded better to immunotherapy. Therefore, the developed nomogram has a high prognostic predictive value.

**Conclusion:** The SUMOylation patterns can well discriminate the tumor microenvironment features of lung adenocarcinoma, especially the immune cell infiltration status. The SUMOylation score can further assess the relationship between SUMOylation and immune cell crosstalk and has significant prognostic value and can be used to predict immunotherapy and chemotherapy response in patients with lung adenocarcinoma.

## 1 Introduction

Lung cancer is among the most commonly diagnosed cancers that pose a critical threat to public health (Siegel et al., 2022) and is a leading cause of cancer-related deaths worldwide (Minguet et al., 2016; Horn et al., 2017), approximately 85% of lung cancers are Non-small cell lung cancer with lung adenocarcinoma being the predominant pathological type. Increasing evidence suggests that the tumor microenvironment (TME) plays a critical role in local drug resistance, immune escape, cancer metastasis, and recurrence. It is well known that macrophage M2 type polarization, T regulatory (Treg) cells and MDSC cells can facilitate the advancement of lung adenocarcinoma, and Th2 helper cells can help the tumor immune escape process, however, CD8^+^ T cells and cytotoxic T cells, etc. are the main force of anti-tumor immunity (Anderson and Simon, 2020). Understanding the tumor microenvironment has profound relevance in preventing metastasis, surmounting acquired drug resistance, and boosting therapeutic efficacy.

SUMOylation is one of the post-translational protein modifications and participates in the regulation of the cell cycle, DNA replication and repair, immunomodulation, and other biological processes (Eifler and Vertegaal, 2015; Chang and Yeh, 2020). Five categories of regulatory genes, including SUMO isoforms, SUMO-Activating Enzyme, SUMO-Conjugating Enzyme, SUMO E3 ligases, and SUMO proteases, regulate the homeostasis of SUMOylation and deSUMOylation (Bawa-Khalfe and Yeh, 2010; Chang and Yeh, 2020). SUMOylation protein modification is associated with Myc-driven tumor heterogeneity (Kessler et al., 2012). SENP7 has been reported to sustain the metabolic fitness and effector functions of tumor-infiltrating CD8^+^ T cells (Wu et al., 2022). Overexpression of *SENP1* has also been shown to be involved in lung adenocarcinoma progression (Wang et al., 2013), and *SAE1*, *UBC9*, and *SENP3* are remarkably upregulated in lung adenocarcinoma and are associated with poor prognosis (Han et al., 2010). Recent studies suggest that the dynamic regulatory processes of SUMOylation and deSUMOylation may modulate antitumor immune processes in the TME by targeting multiple immunocytes. PIAS3 inhibits the activated STAT3 signaling pathway to engage in antitumor immune regulation (Zou et al., 2020). The overexpression of SUMO2 increased IL6 production by T cells, which are potent killers of tumor cells (Won et al., 2015). Decreased UBC9 expression suppressed Treg activation and the production of immune inhibitory molecules including IL10, CTLA4, PD-1, and ICOS (Shevach, 2009). AK-981, an inhibitor of SUMOylation, can directly stimulate T cells and enhance T cell sensitivity as well as response to antigens *in vitro* and *in vivo* (Lightcap et al., 2021). In this study, we explored the critical roles of SUMOylation in lung adenocarcinoma TME.

Several studies have revealed that SUMOylation regulatory genes are involved in the process of TME regulation (Chang and Ding, 2018). However, these studies are limited in that they study individual SUMOylation regulators, and a comprehensive analysis of the effect of SUMOylation on the tumor immune microenvironment has not been conducted before. By identifying different SUMO-associated TME types modulated by multiple SUMOylation regulators, we can gain a more comprehensive understanding of the regulation of the lung adenocarcinoma tumor microenvironment by SUMOylation. Therefore, we developed and validated SUMOylation patterns and signature to predict tumor microenvironmental phenotype and prognostic risk in lung adenocarcinoma.

## 2 Materials and methods

### 2.1 Data source and processing

Lung adenocarcinoma datasets GSE31210 (*N* = 226) (Okayama et al., 2012), GSE37745 (*N* = 106) (Botling et al., 2013), GSE50081 (*N* = 180) (Der et al., 2014), and GSE72094 (*N* = 398) (Schabath et al., 2016) were downloaded from the NCBI gene expression omnibus (GEO) database. The samples with missing survival data were removed and all data were log2 (x+1) normalized and corrected. The ComBat method in the “SVA” package was used to eliminate batch effects among the four GEO datasets and to integrate the four GEO datasets for subsequent analyses. All datasets contained a total of 857 lung adenocarcinoma sample data containing clinical information such as survival data, gender, age, pathological stage, and smoking history. The lung adenocarcinoma transcriptome expression dataset was downloaded through the cancer genome atlas (TCGA) database containing a total of 515 lung adenocarcinoma samples (different samples from the same patient were homogenized by taking the mean value) and 59 normal tissue samples; all expression data were downloaded in the fragments per kilobase of exon per million mapped fragments format and converted to the transcripts per kilobase million (TPM) format with log2 (TPM+1) data normalization operation. The survival data of 500 tumor samples was known, of which the data of samples with a survival time of 0 was removed and all others were rounded to survival time in years. Mutation data and copy number variation (CNV) data for lung adenocarcinoma were downloaded from the TCGA Data Portal (https://tcga-data.nci.nih.gov/tcga/).

### 2.2 Unsupervised consensus clustering for the identification of SUMOylation patterns

By reviewing the literature related to SUMOylation, we identified 33 genes recognized as critical regulators in SUMOylation (Chang and Yeh, 2020). Subsequently, we performed unsupervised consensus clustering using the “ConsensusClusterPlus” package based on the mRNA expression of the 33 SUMOylation regulators. We use the “km” clustering algorithm, the distance calculation algorithm is “euclidean,” and the random seed is set to “123,456". The optimal clusters were determined under the cumulative distribution curve (CDF), and the rationality of the clusters was further verified using principal component analysis (PCA). Consensus clustering was adopted with 1,000,000 replicates to optimize clustering results. The clustering method for unsupervised consensus clustering analysis based on the expression of differentially expressed key SUMOylation genes is consistent with the above.

### 2.3 Gene set variation analysis and biological pathways

We used the R package GSVA (Hanzelmann et al., 2013) to study the biological processes associated with different SUMOylation modification patterns. Hallmark gene sets (Liberzon et al., 2015) and well-defined gene sets of biological features were downloaded from MsigDB (http://www.gsea-msigdb.org/gsea/msigdb/). The gene set of tumor-associated biological pathways was were obtained from the compilation by Mariathasan et al. (Mariathasan et al., 2018) and included the following: angiogenesis, immune checkpoint, cell cycle regulators, Pan fibroblast transforming growth factor beta (Pan F TBRs), epithelial-mesenchymal transition processes involved in EMT1, EMT2, EMT3, and the cell cycle. The immune checkpoint genes (including immune co-stimulatory and immune co-inhibitory molecules) and MHC molecules were derived from the immune-related gene set compiled by Rooney et al. (Rooney et al., 2015).

### 2.4 Gene ontology (GO) annotation, GO enrichment analysis, and KEGG enrichment analysis

The Gene Ontology (GO) and Kyoto Encyclopedia of Genes and Genomes (KEGG) databases are used to annotate genes and their RNA or protein products to determine their unique biological properties. GO annotation and KEGG annotation of SUMOylation-related genes was performed using the R package “clusterProfiler” with a cutoff value of false discovery rate (FDR<0.05).

### 2.5 Estimation of immune cell infiltration by ssGSEA and deconvolution algorithm

The gene set for each immune cell-specific marker was compiled from a recent study, and Single Sample Gene Set Enrichment Analysis (ssGSEA) (Barbie et al., 2009) was used to quantify the relative abundance of 24 immune cell types in the TME and was expressed as an enrichment fraction (Bindea et al., 2013). Subsequently, the EPIC immune infiltration algorithm (Racle et al., 2017) and the TIMER immune algorithm (Li et al., 2017) were used to calculate the extent of infiltration of each immune cell in lung adenocarcinoma tissue. Tumor tissue with abundant immune cell infiltration is consistent with a higher immune score and lower tumor purity.

### 2.6 Identification of differentially expressed genes (DEGs) associated with SUMOylation patterns and the construction of their protein-protein interaction (PPI) networks

The R package “limma” was used to measure DEGs associated with different SUMOylation patterns in lung adenocarcinoma samples, with the significance filtering criteria for DEGs of |FC| > 1.5 and FDR <0.05. The interaction of DEGs was investigated using the STRING database and used to build PPI networks, and the screening network type was a physical subnetwork with a confidence score ≥0.7. We used the “CytoHubba” plugin (Chin et al., 2014) in “Cytoscape” software (Shannon et al., 2003) to calculate the number of neighboring nodes in the PPI network, and filtered genes with neighboring nodes ≥5 as SUMOylation-related key genes.

### 2.7 Calculation of SUMOylation scores

We performed a univariate cox regression analysis using DEGs associated with SUMOylation modification patterns. Next, we screened the significant prognostic correlates (*p* < 0.05) among them for the optimal variables for constructing SUMOylation scores using the least absolute shrinkage and selection operator (LASSO) regression method. The SUMO score was calculated as follows: RiskScore = Exp (Gene1)*β1+Exp (Gene2)*β2+. . . + Exp (Genen)*βn. Where Exp is the expression of the genes and *β* is the regression coefficient calculated by LASSO. Patients were then divided into the high-risk and low-risk groups according to the optimal cut-off value calculated using the surv-cutpoint function in the R package “survival”. Prognostic analyses were conducted using the R package “survminer”. Receiver operating characteristic (ROC) curves were used to evaluate the prognostic performance of the SUMOylation score and the nomogram model. The area under the curve (AUC) was calculated using the R package “timeROC,” while multiple regression analysis was used to verify the validity of the score as an independent prognostic factor for lung adenocarcinoma.

### 2.8 Response to immunotherapy and sensitivity to chemotherapeutic substances

The immunotherapy cohort GSE126044 (Cho et al., 2020) contained a total of 16 lung adenocarcinoma recipients of nivolumab (anti-PD1 drug) treatment, five of which were responsive to (CR and PR) and 11 non-responsive to immunotherapy (PD and SD). (Tumor Immune Dysfunction and Exclusion) TIDE (http://tide.dfci.harvard.edu/) is applied to predict immunotherapy response for lung adenocarcinoma patients. Gene expression data from pretreatment tissue samples were collated and transformed into TPM format with log2 (TPM+1) normalization for further analysis. Chemotherapy drug sensitivity analysis was performed using the R package “pRRophetic” (Geeleher et al., 2014) to predict clinical response to chemotherapy.

### 2.9 Clinical samples, RNA extraction, and quantitative real-time PCR

Eighteen pairs of LUAD tissues and adjacent non-cancerous lung tissues were obtained from the First Affiliated Hospital of Soochow University after informed consent from patients. Pathological diagnostics for patients with LUAD were assessed according to the Revised International System for Staging Lung Cancer. All patients included in this study did not receive any radiation therapy, chemotherapy, or immunotherapy. The experiment was approved by the Academic Advisory Board of Soochow University.

Total RNA was isolated using Trizol reagent (Beyotime, China) and reverse transcribed into cDNA by Hiscript III Reverse Transcriptase (Vazyme, China). Real-Time PCR was performed using ChamQ SYBR qPCR Master Mix (Vazyme, China) on a LightCycler96 real-time PCR system (Roche, Switzerland). The specific primers we used are listed in Supplementary Table S1.

### 2.10 Statistical analysis

All statistical analyses in this study were performed using the R software 4.1.2 or GraphPad Prism 9. For quantitative data, normally distributed variables were analyzed using the Student’s t-test, and non-normally distributed variables were analyzed using the Wilcoxon rank-sum test. Statistical significant differences between three and more data sets were analyzed using the Kruskal–Wallis method for non-parametric statistical tests and one-way ANOVA for parametric statistical tests. We calculated correlation coefficients using Spearman and distance correlation analysis. Survival analyses were conducted using the Kaplan–Meier method and log-rank tests, while Cox proportional risk regression models were used to analyze the relationship between SUMOylation patterns and regulatory genes and prognosis. All statistical comparisons in this study were two-sided with *α* = 0.05 and the Benjamini-Hochberg method was employed to control the false discovery rate (FDR) for multiple hypothesis testing. *p < 0.05, **p < 0.01, ***p < 0.001.

## 3 Results

### 3.1 Genetic variation in SUMOylation regulatory genes in lung adenocarcinoma

**FIGURE 1:**
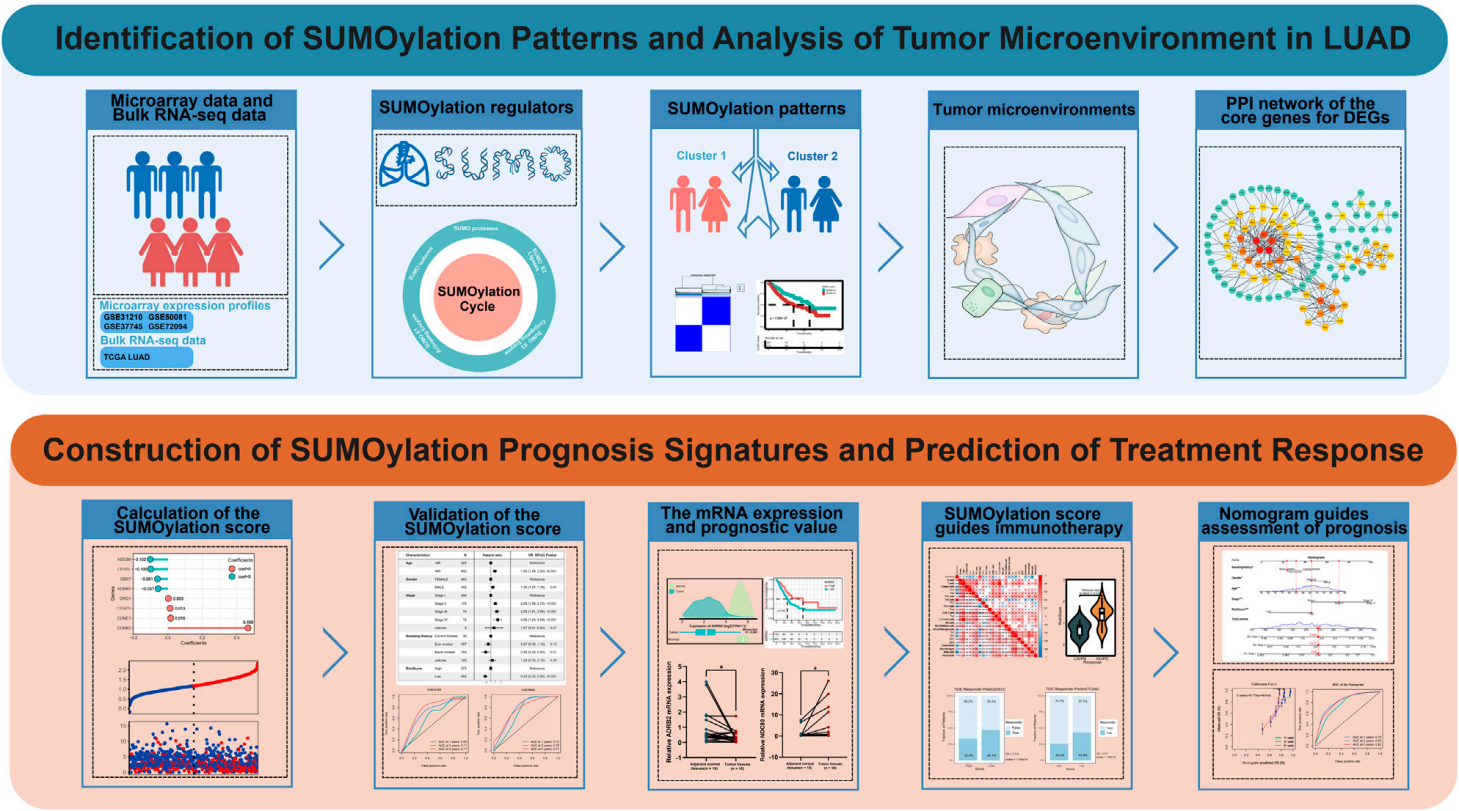
Flow chart of our study.

**FIGURE 2:**
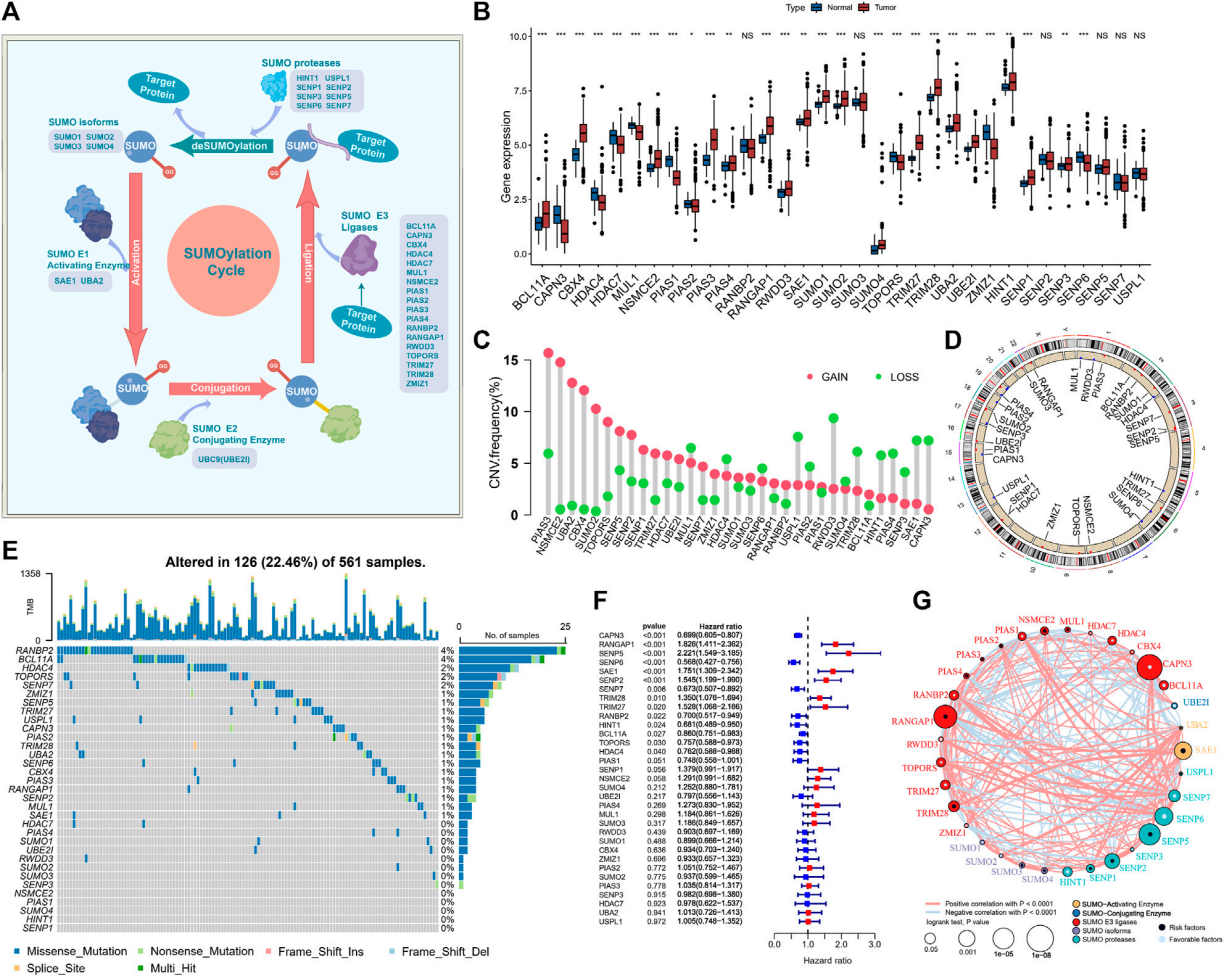
SUMOylation regulatory gene landscape in lung adenocarcinoma. **(A)** The reversible process regulation of SUMOylation and deSUMOylation. **(B)** Differential expression of SUMOylation regulatory genes among lung adenocarcinoma and normal sample tissues. **(C)** Frequency of CNV (copy number variation) of SUMOylation regulatory genes in lung adenocarcinoma. **(D)** Chromosomal localization of the CNV of SUMOylation regulatory genes. **(E)** The landscape of tumor somatic mutations in SUMOylation regulatory genes in lung adenocarcinoma. **(F)** Univariate cox prognostic analysis of SUMOylation regulatory genes in lung adenocarcinoma. **(G)** Interaction of SUMOylation regulatory genes in lung adenocarcinoma.

The flow chart shows our research procedure (Figure 1). We investigated the role of 33 SUMOylation-modified regulatory genes in lung adenocarcinoma, namely, SUMO isoforms (*SUMO1*, *SUMO2*, *SUMO3*, *SUMO4*), SUMO-Activating Enzyme (*SAE1*, *UBA2*), SUMO-Conjugating Enzyme (*UBE2I*), SUMO E3 Ligases (*BCL11A*, *CAPN3*, *CBX4*, *HDAC4*, *HDAC7*, *MUL1*, NSMCE2, PIAS1, PIAS2, PIAS3, PIAS4, RANBP2, *RANGAP1*, *RNF212*, *RWDD3*, *TOPORS*, *TRIM27*, *TRIM28*, *ZMIZ1*), SUMO proteases (*HINT1*, *SENP1*, *SENP2*, *SENP3*, *SENP6*, *SENP5*, *SENP7*, *USPL1*). The reversible process of SUMOylation is regulated by the aforementioned regulatory genes (Figure 2A). We first performed GO and KEGG enrichment analysis of the 33 SUMOylation regulatory genes. GO enrichment revealed that the 33 SUMOylation-regulated genes were remarkably enriched in biological processes such as lysine modification, protein SUMOylation, regulation of small protein covalent protein modification (Supplementary Figure S1A), and molecular functions such as ubiquitinated protein convertase activity, SUMO convertase activity, among others (Supplementary Figure S1B). KEGG enrichment analysis revealed other SUMOylation-related pathways (Supplementary Figure S1C).

Subsequently, for the comprehensive understanding of the SUMOylation regulatory genes, we performed the Spearman correlation analysis of the 33 SUMOylation regulatory genes to assess their pattern of co-expression (Supplementary Figure S1D) and found a broad significant negative correlation between the expression of *CAPN3* and *PIAS1* and other regulatory genes, as well as a significant positive correlation between *CAPN3* and *PIAS1* expression. Notably, most of the SUMOylation regulatory genes were remarkably upregulated in tumor tissues, and overall, the expression of SUMOylation regulatory genes was remarkably different between the tumor and normal samples (Figure 2B). PCA based on paired tumor and normal samples identified the SUMOylation regulatory genes whose expression was significantly different between normal and tumor samples (Supplementary Figure S1E).

Moreover, CNVs were very common in the 33 regulatory genes; CNV amplifications were prevalent in *PIAS3*, *NSMCE2*, *UBA2*, *CBX4*, and *SUMO2,* while CNV deletions were prevalent in *RWDD3*, *USPL1*, *SAE1*, and *CAPN3* (Figure 2C). The CNV occurred widely in genes on a variety of chromosomes (Figure 2D); however, they were concentrated mainly on chromosomes 1, 3, 4, 6, 12, 15, and 19. We further evaluated the prevalence of somatic mutations in SUMOylation regulatory genes. Of the 561 lung adenocarcinoma samples, 126 (22.46%) had alterations, mainly missense mutations and non-sense mutations, in SUMOylation regulatory genes. *RANBP2* and *BCL11A* had the highest mutation frequency of 4%, followed by *HDAC4*, *TOPORS,* and *SENP7* with 2% mutation frequency; most mutations occurred in the regulatory genes SUMO activator and E3 ligase, and less in SUMO isoforms (Figure 2E). Subsequently, we analyzed the mutational co-occurrence of the SUMOylation regulatory genes and found that SUMO2 co-occurred with mutations in both RANGAP1 and PIAS4 (Supplementary Figure S1F).

Furthermore, to determine the relationship between the 33 SUMOylation regulatory genes and the prognosis of lung adenocarcinoma patients, we performed a cox risk proportional regression model was used to determine the relationship between the 33 SUMOylation regulatory genes and the prognosis of lung adenocarcinoma patients, and the forest plot revealed that *CAPN3*, *SENP6*, *SENP7*, *RANBP2*, *HINT1*, *BCL11A*, *TOPORS*, and *HDAC4* were protective factors for lung adenocarcinoma and were generally downregulated, while *RANGAP1*, *SENP5*, *SAE1*, *SENP2*, *TRIM28*, and *TRIM27* were risk factors for lung adenocarcinoma and were upregulated in lung adenocarcinoma (Figure 2F). The multivariate Cox proportional Hazards regression analysis further established that SUMOylation regulatory genes were significantly associated with lung adenocarcinoma prognosis (Supplementary Figure S1G). In conclusion, we mapped the relationship between SUMOylation regulatory genes and lung adenocarcinoma prognosis (Figure 2G). In summary, we determined that SUMOylation regulatory genes differed significantly between normal and lung adenocarcinoma tissues and were also markedly correlated with the frequency of mutations and CNVs in lung adenocarcinoma. In addition, we demonstrated that altered expression and genetic variation of specific SUMOylation regulatory genes have a critical role in the development and prognosis of lung adenocarcinoma.

### 3.2 Identification of two different SUMOylation patterns based on the expression of 33 SUMOylation-regulated genes

**FIGURE 3:**
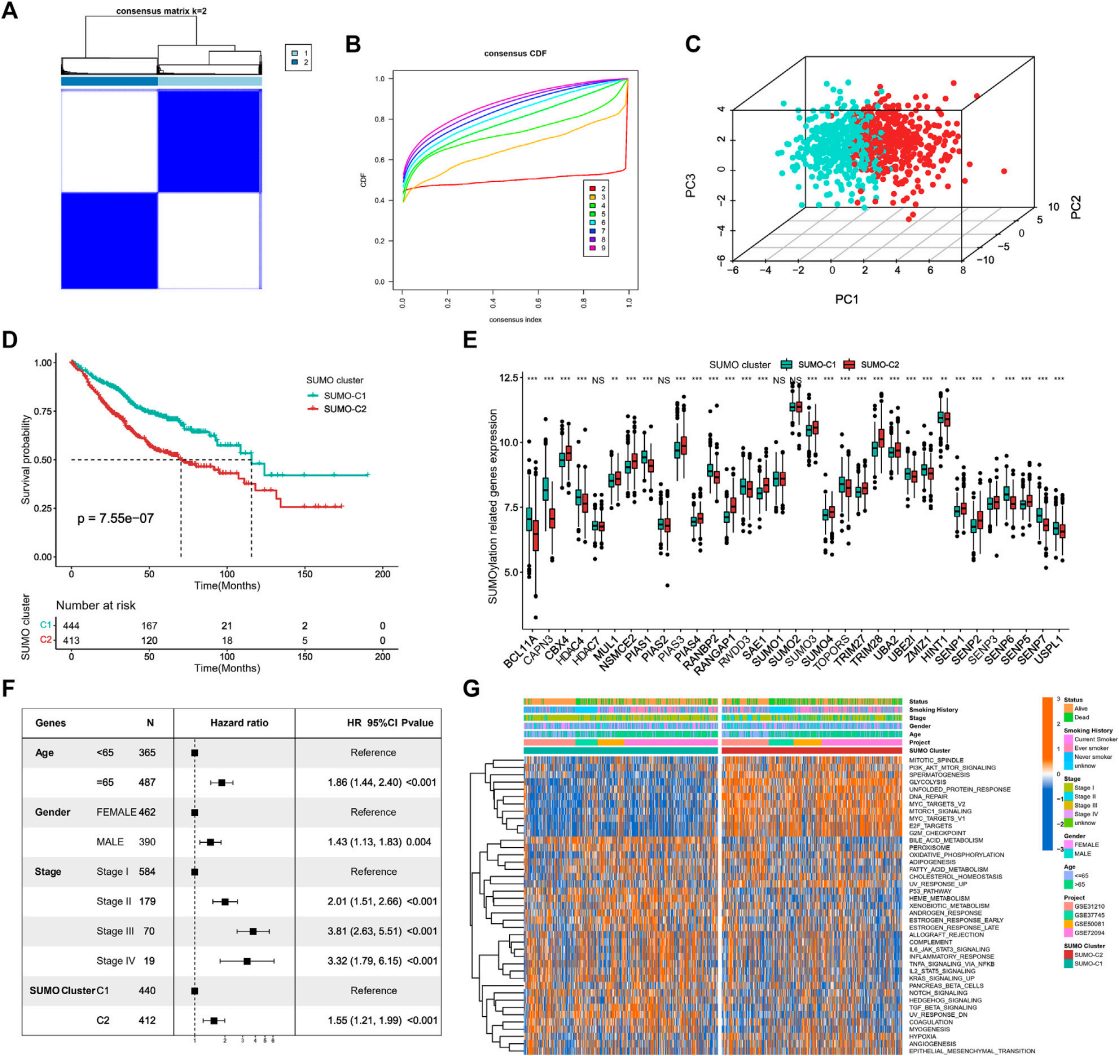
Identification of SUMOylation patterns and their associated biological functions. **(A)** Unsupervised cluster analysis based on 33 SUMOylation regulatory genes, K = 2. **(B)** The cumulative distribution curve (CDF) suggests that the optimal K value for unsupervised clustering is 2. **(C)** Principal component analysis (PCA) based on mRNA expression data of SUMOylation regulatory genes. **(D)** Kaplan-Meier curves for overall survival OS of two SUMOylation patterns for a total of 857 lung adenocarcinoma samples (including 444 SUMO-C1 and 413 SUMO-C2 cases). (Log-Rank test *p* = 7.55e-07). **(E)** SUMOylation regulatory genes were significantly differentially expressed between the two SUMOylation models. **(F)** SUMOylation patterns can be an independent prognostic factor for lung adenocarcinoma. **(G)** Heatmap showing the GSVA scores of the biological pathways of the HALLMARK gene set among two SUMOylation patterns in lung adenocarcinoma. Annotated with Status, Smoking History, Stage, Gender, Age, GEO cohort, SUMOylation patterns. Orange is high expression and blue is low expression.

For exploring different SUMOylation patterns, we collated 857 lung adenocarcinoma patients in four datasets of the GEO database for unsupervised consistency clustering based on 33 SUMOylation regulatory genes. Based on the variation of the area under the CDF curve (Figures 3A, B; Supplementary Figure S2A), we determined the optimal number of clusters to be 2 (K = 2). We subsequently performed PCA of the two SUMOylation patterns based on mRNA expression data of the regulatory genes (Figure 3C) and found that the two different modification patterns could be completely distinguished by SUMOylation regulatory genes. Meanwhile, Kaplan–Meier survival analysis revealed that patients with the SUMO-C2 pattern showed a worse prognostic survival (log-Rank test p = 7.55e-07) (Figure 3D). The occurrence of the SUMO-C2 pattern was consistent with the downregulation of BCL11A, CAPN3, HDAC4, PIAS4, UBA2, UBE2I, SENP6, SENP7, and USPL1, and the upregulation of CBX4, NSMCE2, PIAS3, RANGAP1, TRIM28 and SENP2 (Figure 3E). The Heatmap further confirmed that the expression of the 33 SUMOylation-regulated genes was significantly different in the two SUMOylation patterns (Supplementary Figure S2B). The multivariate Cox regression results indicated, in agreement with Kaplan–Meier results, that the SUMOylation patterns can be an independent prognostic factor, and compared with SUMO-C1, SUMO-C2 was associated with poor prognostic survival, HR = 1.55, p < 0.001 (Figure 3F).

To further explore the differences in key biological pathways associated with the two SUMOylation patterns. The GSVA algorithm was used to calculate the Hallmark gene set scores and convert them into a scoring matrix of the gene set. We found that there were significant differences in GSVA scores of Hallmark gene sets consistent with SUMO-C1 and SUMO-C2 (Figure 3G), and SUMO-C2 were mainly enriched in biological pathways related to the cell cycle, glucose metabolism, and genetic material replication and repair, such as G2M checkpoint, E2F targets, DNA repair, Glycolysis, and PI3K-AKT-mTOR signaling pathways and was highly active in biological processes related to cancer progression such as hypoxia, angiogenesis, and epithelial-mesenchymal transition (Supplementary Figure S2C). In contrast, SUMO-C1 was mainly enriched in tumor immune-related processes, such as TNFA signaling via NF-κB, IL6-STAT3 signaling, complement, inflammatory response, and IL2-STAT5 signaling, allograft rejection signaling pathway. This suggests that the SUMOylation pattern based on SUMOylation regulatory genes may influence the immune microenvironment status. Biological mechanisms for the poor prognosis associated with SUMO-C2 maybe because of the active immune microenvironment of the SUMO-C1 and the active stromal microenvironment of the SUMO-C2. SUMO-C1 may present better antitumor effects and better prognostic survival in lung adenocarcinoma through high activation of antitumor immunity and inhibition of the pro-cancer progressive stromal microenvironment.

### 3.3 Different SUMOylation patterns are associated with varying immune microenvironments

**FIGURE 4:**
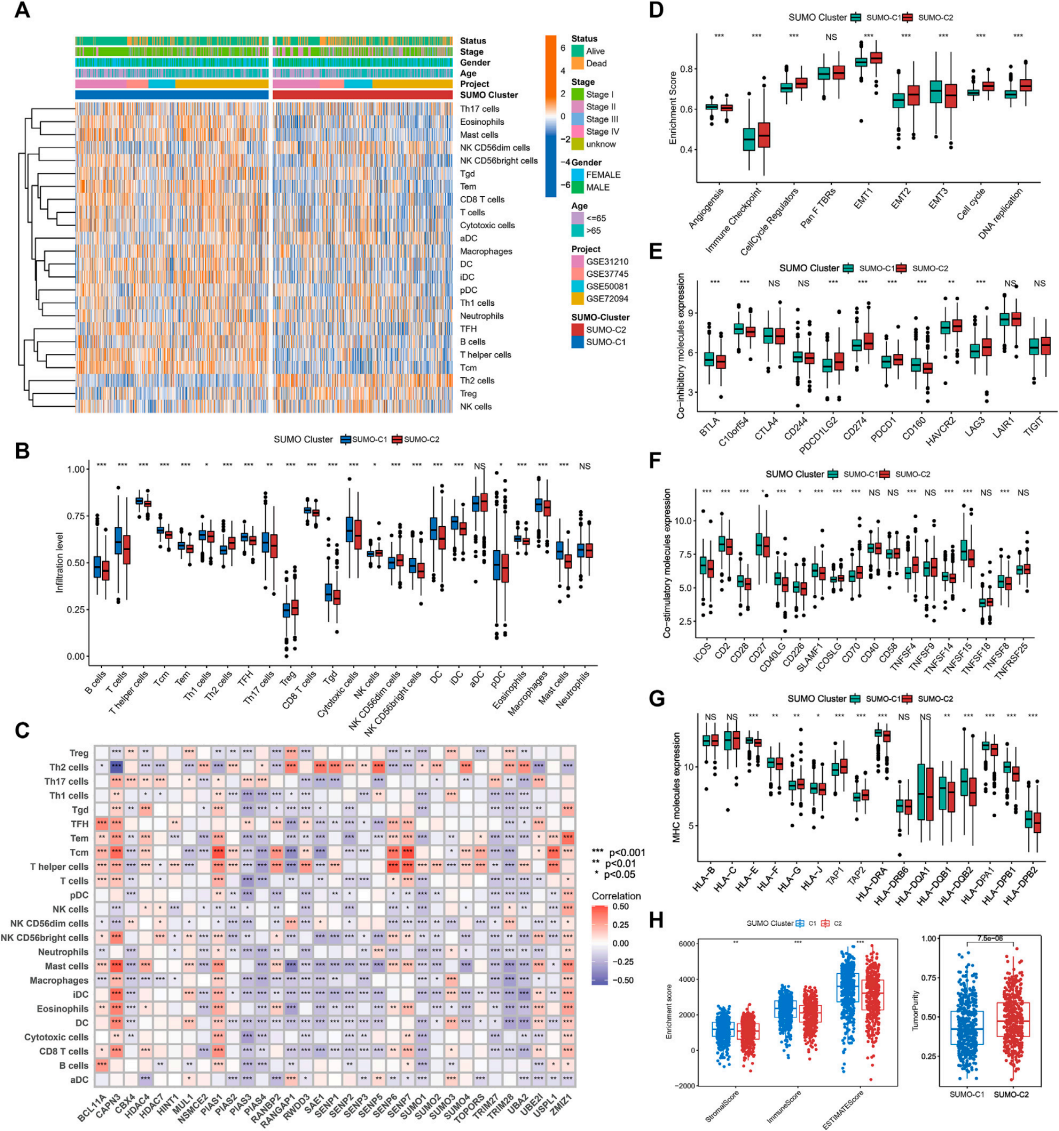
The tumor microenvironment differs significantly between SUMOylation patterns. **(A)** The heatmap shows the GSVA scores of 24 immune cells among two SUMOylation patterns in lung adenocarcinoma. Annotated with Status, Smoking History, Stage, Gender, Age, GEO cohort, and SUMOylation patterns. Orange is high expression and blue is low expression. **(B) **Wilcoxon test for differences in GSVA scores of 24 immune cells in two SUMOylation models. **(C)** Correlation between 33 SUMOylation regulatory genes and immune infiltrating cells using Spearman analysis. **(D)** Differences in oncogenic matrix microenvironment between two SUMOylation patterns. **(E)** Significantly differential expression of co-inhibitory molecules between the two SUMOylation patterns. **(F)** Differential expression of co-stimulatory molecules and **(G)** MHC molecules between two SUMOyaltion patterns. **(H)**Wilcoxon test for differences in immune scores, stromal scores, and tumor purity between the two SUMOylation models.

To explore the potential mechanisms by which SUMOylation patterns regulate the immune microenvironment, we used the ssGSEA algorithm to calculate the abundance of 24 different immune cells. A heatmap was used for visualizing the difference in immune cell infiltration (Figure 4A), and the Wilcoxon rank-sum test was used for comparing the discrepancy in immune cell infiltration consistent with SUMO-C1 and SUMO-C2 patterns (Figure 4B). Notably, SUMO-C1 showed a widespread active state of immune cells, while SUMO-C2 showed an inactive state of immune cells. The infiltration of B cells, T cells, T helper cells, Central Memory T cell (Tcm), Effector memory T cells (Tem), Follicular helper T cell (TFH), Th17 cells, CD8 T cells, cytotoxic cells, dendritic cells (DCs), and mast cells were significantly higher in the SUMO-C1 than SUMO-C2 group. In contrast, immune cells such as Th2 cells and Treg cells were significantly activated in SUMO-C2. Thereafter, we calculated immune cell infiltration levels in lung adenocarcinoma using the EPIC algorithm and TIMER algorithm to further support the differences in immune cell infiltration levels and compared the significance of the differences between the SUMO-C1 and SUMO-C2 groups using the Wilcoxon rank-sum test. We found that the SUMO-C1 group exhibited higher levels of immune cell infiltration, particularly CD8 T cells, which has been reported to have a killing effect on tumor cells thereby producing protective immunity against tumors (Supplementary Figures S3A, S3B). Furthermore, Spearman correlation between each SUMOylation regulatory gene and immune cell revealed that *CAPN3* was widely associated with various immune cells such as CD8+T cells (r = 0.32), DCs (r = 0.343), iDCs (r = 0.432), Mast cells (r = 0.468), Tcm (r = 0.421), Tem (r = 0.275), TFH (r = 0.291), Th2 cells (r = 0.563), and Treg (r = 0.115), indicating that *CAPN3* among SUMOylation regulatory genes is closely associated with immune cell infiltration in the tumor microenvironment of lung adenocarcinoma. Overall, the immune microenvironment was regulated by SUMOylation regulatory genes in lung adenocarcinoma mainly by modulating the levels of immune cells such as CD8+T cells, mast cells, T helper cells, Tcm, Tem, and Th2 cells (Figure 4C; Supplementary Figures S3A).

The above analysis supports that SUMO-C1 represents a type of TME with an immune characteristic of tumor suppression. Nevertheless, SUMO-C2 exhibited predominantly pro-cancer biological processes, as well as promoted the stromal microenvironment according to our previous GSVA enrichment analysis of the Hallmark gene set. Furthermore, we assessed the enrichment scores for angiogenesis, immune checkpoint, cell cycle regulation, Pan F TBRS, EMT, and DNA replication using the ssGSEA method. The ssGSEA results showed pathways that can promote immune escape such as EMT1, EMT2, and immune checkpoint pathways were remarkably activated in the SUMO-C2 group (Figure 4D). Moreover, the SUMO-C1 group showed a higher expression of co-stimulatory and MHC molecules and lower expression of co-inhibitory molecules (Figures 4E–G), such as CD274, PDCD1, and LAG3, which are now commonly used as immunotherapeutic checkpoints in lung adenocarcinoma. Finally, we assessed the immune score, stroma score, and tumor purity score using the Estimate package for lung adenocarcinoma expression data and found that SUMO-C1 had a better immune score as well as a higher stroma score (Figure 4H), while SUMO-C1 had a lower tumor purity score, which further explains its association with better prognosis. Altogether, the above analysis demonstrated that the SUMO-C2 SUMOylation pattern promoted the development of a silent anti-tumor immune microenvironment and an active tumorigenic stromal microenvironment.

### 3.4 PPI of SUMOylation pattern-associated genes (SPAGs) and identification of SUMOylation genomic subtypes

**FIGURE 5:**
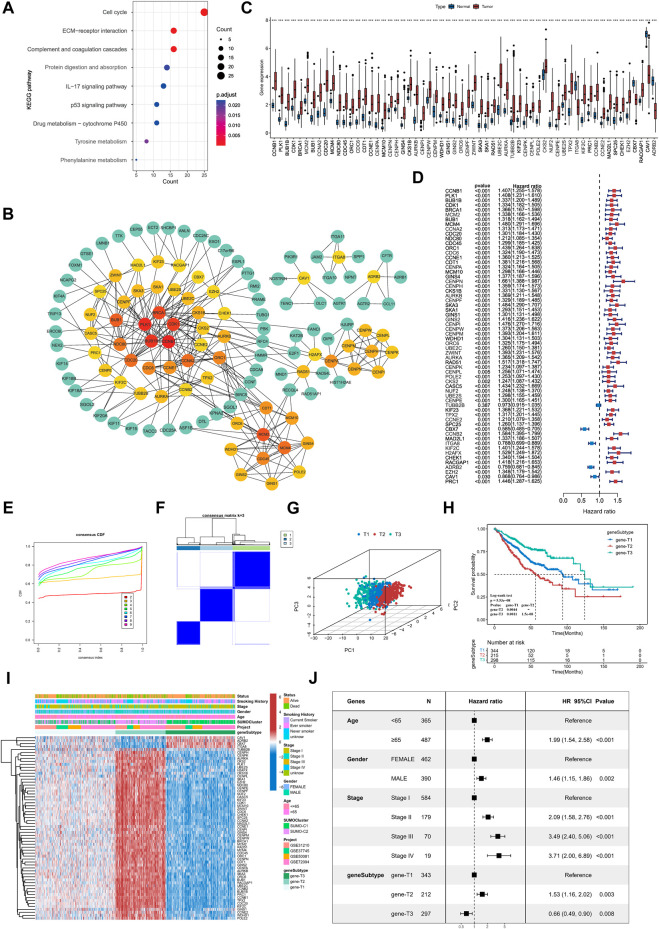
Identification of key genes differentially expressed between SUMOylation patterns and characterization of SUMOylation genomic subtypes. **(A)** Functional annotation of SUMOylation differential genes by KEGG enrichment analysis. **(B)** The Protein-Protein Interaction Network (PPI) of key genes associated with SUMOylation. **(C)** Differential expression of SUMOylation key genes between lung adenocarcinoma and normal tissue samples. **(D)** Prognostic value analysis of SUMOylation key genes for lung adenocarcinoma. **(E)** CDF curves for unsupervised clustering with K = 2 to 9. **(F)** Consensus clustering graph with K = 3. **(G)** PCA of SUMOylation genomic subtypes based on key genes transcriptome. **(H)** Kaplan-Meier curves for overall survival OS for the three SUMOylation genomic subtypes. (Log-Rank test *p* = 5.53e-08, *p*-value less than 0.001 for both comparisons). **(I)** The heatmap shows the identification of 3 different genomic subtypes by unsupervised clustering based on 62 SUMOylation-associated key genes. Annotated with Status, Smoking History, Stage, Gender, Age, SUMOylation patterns, GEO cohort, and genomic subtypes. Red is high expression and blue is low expression. **(J)** Multivariate cox analysis indicates that SUMOylation genomic subtype can be an independent prognostic factor for lung adenocarcinoma.

To further explore the underlying biological processes modulated in different SUMOylation patterns, we identified 906 significant DEGs (|FoldChange|>1.5, FDR<0.05) as SUMOylation pattern-associated genes (SPAGs) using the R package limma. These DEGs were also significantly associated with lung adenocarcinoma prognosis, indicating the critical role of SUMOylation patterns in dictating prognosis. KEGG functional annotation showed significant enrichment of SUMOylation-related genes to biological pathways such as cell cycle-related processes (cell cycle), cell adhesion (ECM-receptor interaction), immune activation signaling pathways (complement and coagulation cascades, IL 17 signaling pathway) (Figure 5A), while GO functional annotations revealed enrichment of biological processes such as regulation of the cell cycle phase transition, cell growth, humoral immune response, cell-cell junction and response to hypoxia (Supplementary Figures S4A–C).

Next, we constructed a protein interaction network (PPI, network type = physical subnetwork, minimum required interaction score = 0.7) of SPAGs using the STRING database and calculated the relationship between nodes using the Cytohubba plugin. We then classified genes with neighboring nodes ≥5 of 62 genes as key SPAGs and mapped their interaction (Figure 5B; Supplementary Figure S4D). Of the key genes, *CCNB1* had the most neighboring nodes, followed by *PLK1*, *BUB1B*, *CDK1*, and *BRCA1*. It has been recently reported that the key genes in the PPI network can represent the functional characteristics of all genes accurately. Differential expression analysis by the TCGA dataset showed that key genes were significantly differentially expressed in lung adenocarcinoma from normal tissue samples (Figure 5C). Meanwhile, key genes extensively indicated significant prognostic significance, and CBX7, ITGA8, ADRB2, and CAV1 were significant favorable factors for lung adenocarcinoma (Figure 5D).

Unsupervised consensus clustering was carried out based on the identified key SPAGs. Three genomic subtypes were obtained, namely, gene-T1, gene-T2, and gene-T3 by CDF curve (Figures 5E, F; Supplementary Figure S4E). PCA suggested significant differences among the three genomic subtypes identified based on key genes (Figure 5G). Kaplan–Meier survival analysis indicated that gene-T2 was associated with the worst, gene-T3 with the best, and gene-T1 with the intermediate prognostic outcome, indicating different clinical courses of the genomic subtypes (Figure 5H). Moreover, the heatmap revealed differential expression of the 62 key genes among the three genomic subtypes (Figure 5I). This is consistent with the results of the prognostic utility of key genes in lung adenocarcinoma, as the heatmap showed that *CAV1*, *ADRB2*, *CBX7*, and *ITGAB8* which were favorable factors for lung adenocarcinoma were remarkably upregulated in gene-T3, and the remaining risk factors for lung adenocarcinoma were significantly upregulated in the gene-T2 subtype. We then combined prognostic features such as age, sex, pathological stage, and genomic subtype with survival information for multicox analysis, which revealed that all the above prognostic features including genomic subtype are independent prognostic factors in lung adenocarcinoma; gene-T2 vs. gene-T1 (HR = 1.53, *p* = 0.003) and gene-T3 vs. gene- T1 (HR = 0.66, *p* = 0.008) (Figure 5J). Finally, we determined that SUMOylation regulatory genes were significantly differentially expressed among the three genomic subtypes and their expression in the gene-T1 subtype was intermediate to that in the gene-T2 and gene-T3 subtypes (Supplementary Figure S4F), indicating that different genomic subtypes further reflect the differences in the SUMOylation patterns.

### 3.5 Calculation and prognostic significance of the SUMOylation score

**FIGURE 6:**
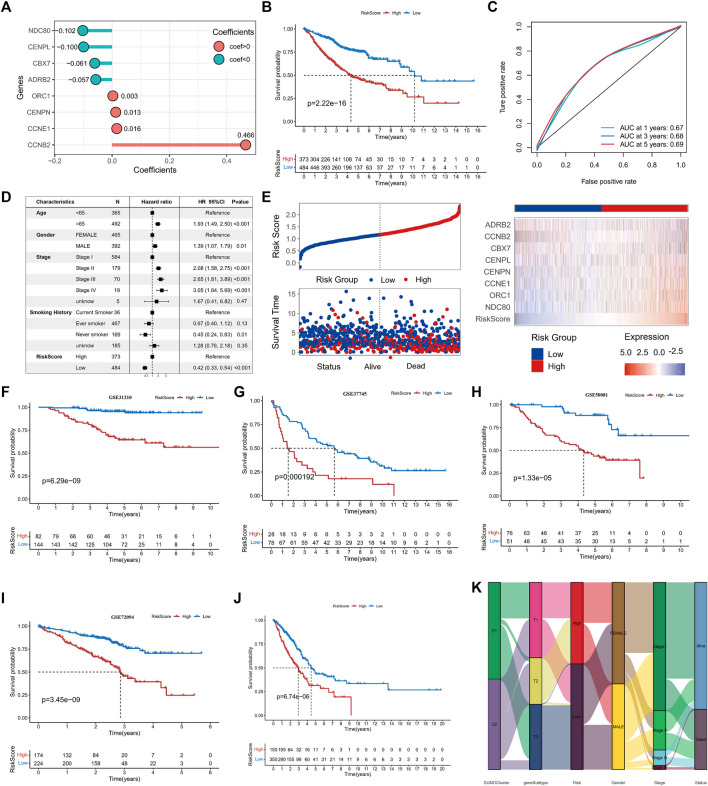
Construction of SUMOylation score and validation of prognostic value. **(A)** LASSO regression coefficients of model genes. **(B)** Kaplan-Meier curves for overall survival (OS) in lung adenocarcinoma patients between high- and low-SUMOylation score. **(C)** Operating characteristic curve for assessing the predictive performance of SUMOylation score for OS of lung adenocarcinoma, with AUCs of 0.67, 0.68, and 0.69 at 1, 3, and 5 years, respectively. **(D)** Multivariate cox analysis indicates that the SUMOylation score can work as an independent prognostic factor for lung adenocarcinoma. **(E)** The risk heatmap illustrates the variations in SUMOylation scores, patient deaths, and model gene expression levels. Red is high expression and blue is low expression. Validation of the prognostic significance of SUMOylation scores in independent datasets GSE31210 **(F)**, GSE37745 **(G)**, GSE50081 **(H)**, GSE72094 **(I)**. **(J)** Validation of significant differences in overall survival (OS) between patients with high- and low SUMOylation scores using the TCGA external dataset. **(K)** The alluvial map reveals the association between SUMOylation patterns, SUMOylation genomic subtypes, SUMOylation score groups, and other clinicopathological prognostic features.

We aimed to determine the prognostic value and biological significance of SUMOylation modification patterns in lung adenocarcinoma and to develop a SUMOylation pattern-based prognostic model for the assessment of individual lung adenocarcinoma patients. For this, we first performed a univariate cox analysis of the 62 key SPAGs along with LASSO regression to screen the best variables. Eventually, eight SUMOylation key genes that were significantly differentially expressed between normal tissues and tissues in lung adenocarcinoma and significantly associated with prognosis in lung adenocarcinoma were screened, and a SUMOylation-based prognostic signature model was constructed based on the expression of these genes and their coefficients (Supplementary Figures S5A, B). The risk score was calculated formulas follows: risk score = Exp*NDC*80*(−0.1024) + ExpORC1*0.0027+ ExpCCNE1*0.0164+ ExpCENPN*0.0127+ ExpCENPL*(−0.0997) + ExpCBX7*(−0.0606) + ExpCCNB2*0.4661+ ExpADRB2*(−0.0568) (Figure 6A). All lung adenocarcinoma samples were divided into high-risk and low-risk groups using the Survival package to calculate the optimal cut-off values. Survival analysis revealed that patients with higher scores were significantly associated with worse prognosis (log-rank *p* = 2.22e-16) (Figure 6B); the 1-year ROC AUC was 0.67, 3-year AUC was 0.68, and 5-year AUC was 0.69, indicating that the risk score based on the SUMOylation model had relatively superior prognostic accuracy compared to the vast array of existing prognostic models (Figure 6C). Multivariate Cox regression analysis incorporating age, gender, pathological stage, smoking history, and risk score confirmed that the risk score was an independent prognostic factor for assessing patient outcomes. (low vs. high, HR = 0.42 (0.33, 0.54), p < 0.001) (Figure 6D). ADRB2 and CBX7 expression were significantly negatively correlated with the risk score (Figure 6E). More deaths were observed in the high-risk group.

Four datasets, GSE31210 (Figure 6F), GSE37745 (Figure 6G), GSE50081 (Figure 6H), and GSE72094 (Figure 6I), and the external dataset TCGA-LUAD (Figure 6J) were used to further validate the stability and prognostic value of SUMOylation scores. The results of survival analysis gave *p*-values <0.001 for log-rank tests in all data sets, suggesting that the SUMOylation model risk score can consistently forecast patient prognostic outcomes; the ROC analysis also showed a high diagnostic value of the risk score in predicting lung adenocarcinoma prognosis (Supplementary Figures S5C–G). Multivariate cox regression analysis of TCGA lung adenocarcinoma cohort incorporating various prognostic factors such as age further confirms that the risk score is an independent risk factor for lung adenocarcinoma (low vs. high, HR = 0.52 (0.38, 0.71), *p* < 0.001) (Supplementary Figure S5H). To better understand the direct relationship between SUMOylation patterns and genomic subtypes, we constructed a Sankey diagram and observed that the vast majority of SUMO-C2 and a small fraction of SUMO-C1 comprise genomic subtype-T1 and then almost all of them comprise the Low Riskscore group (Figure 6K).

### 3.6 Experimental validation for genes of risk model

To verify the expression levels of model genes, we collected tumor tissues from 18 pairs of lung adenocarcinoma patients and the corresponding normal tissues adjacent to the cancer and performed quantitative real-time PCR. We also analyzed the expression levels and prognostic value of the model genes using the TCGA lung adenocarcinoma cohort. NDC80, CENPL, ORC1, CENPN, CCNE1, and CCNB2 were significantly upregulated in the TCGA cohort as well as in the 18 pairs of lung adenocarcinoma patient samples, and high expression was significantly associated with poor prognosis in lung adenocarcinoma. However, CBX7 and ADRB2 were significantly downregulated in lung adenocarcinoma tumor tissues and may serve as favorable prognostic factors for lung adenocarcinoma (Supplementary Figures S6A–H, *p* < 0.05).

SUMOylation score indicates immune microenvironment status and immunotherapy response in lung adenocarcinoma.

**FIGURE 7:**
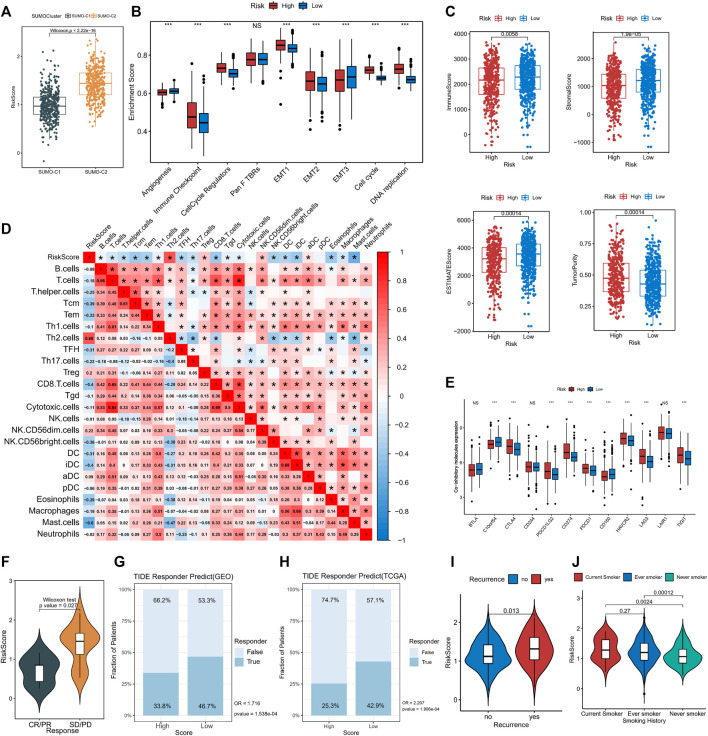
The association of SUMOylation score with tumor microenvironment and clinical characteristics. **(A)** The Wilcoxon test for significant differences in SUMOylation scores between the two SUMOylation patterns. **(B)** Oncogenic matrix microenvironment differences between high- and low-SUMOylation score. **(C)** Significant differences in ImmuneScore, StromalScore, ESTIMATEScore, and TumorPurity between different SUMOylation scores. **(D)** The correlation between SUMOylation score and immune cell infiltration. **(E)** Analysis of differential expression of co-inhibitory molecules between high- and low-SUMOylation score. **(F)** Comparison of SUMOylation scores between groups with and without immunotherapy response. Prediction of immunotherapy response in high and low-risk groups in GEO cohort **(G)** and TCGA cohort **(H)** based on TIDE algorithm. Association of lung adenocarcinoma recurrence **(I)** and smoking history **(J)** with SUMOylation score.

Comparison by the Wilcoxon rank-sum test showed that SUMO-C2 was associated with a higher risk score (Figure 7A), gene-T1 with an intermediate, gene-T2 with the highest, and gene-T3 with the lowest risk score (Supplementary Figure S7A), implying that differences in high and low-risk scores could reflect prognostic differences attributable to different SUMOylation patterns as well as different genomic subtypes. To further determine the relationship between SUMOylation scores and immune infiltration and other biological processes, the differences in the tumorigenic microenvironment between the groups with high- and low-risk scores were investigated. Immune checkpoint, cell cycle regulators, EMT1, EMT2, the cell cycle, and DNA replication were significantly active in the high-risk group (Figure 7B). Assessment of the level of immune infiltration in the Estimate package revealed significantly lower immune scores and higher tumor purity in the high-risk than in the low-risk group (Figure 7C). Moreover, Spearman correlation analysis exhibited a significant negative correlation between SUMOylation score and immune score, and a significant positive correlation between SUMOylation score and tumor purity (Supplementary Figure S7B), reinforcing the accuracy of the assessment of active biological processes in different SUMOylation patterns through risk scores.

Subsequently, Spearman analysis was then used to investigate the correlation between risk score and different immune cell subpopulations, and risk score was overall significantly negatively correlated with the levels of various immune cells. The risk score was significantly positively correlated with Th2 cells (r = 0.68) and Treg cells (r = 0.2)., and was negatively correlated with immune cells such as CD8+T cells (r = −0.4), Tem (r = −0.33), Tcm (r = −0.39), TFH (r = −0.31), CD56 (bright) natural killer cells (r = −0.36), DCs (r = −0.31), (immature Dendritic Cells)iDC (r = −0.4) and mast cells (r = −0.6). In addition, there was a close correlation between the degree of infiltration of Th1 cells, CD8^+^ T cells, T cells, and cytotoxic cells, as well as that of macrophages and iDCs in lung adenocarcinoma. (Figure 7D). These findings suggest that SUMOylation regulatory genes are involved in immune cell infiltration and immune regulation of lung adenocarcinoma tumors. Of these, Th2, Treg, and CD8^+^ T cells, and mast cells are involved in immune dynamic regulation (Supplementary Figure S7C). We then assessed immune checkpoint expression between the high- and low-risk groups to further investigate the effect of SUMOylation on immunotherapy response and found that the expression of clinically common immune checkpoint co-inhibitory molecules such as CD274, PDCD1, CTLA4, PDCD1LG2, HAVCR2, LAG3, and TIGIT was increased in the high-risk group (Figure 7E), while co-stimulatory molecules, such as CD2, CD28, and CD40LG, were decreased in the high-risk group (Supplementary Figure S7D), indicating that SUMOylation scores broadly modulate immune checkpoint expression. In addition, SUMOylation also suppresses the activation of the immune microenvironment by reducing the expression of MHC molecules (Supplementary Figure S7E).

A subsequent analysis of the role of SUMOylation patterns on immunotherapy response using the immunotherapy cohort reported in the lung cancer dataset GSE126044 indicated that the responder group (CR/PR) had low-risk scores and the non-responder group had high-risk scores (Figure 7F), explaining the worse immunotherapy response in high-risk patients. Meanwhile, we evaluated the immunotherapy response of lung adenocarcinoma patients in both high and low SUMOylation score groups in the GEO cohort (High-risk vs. Low-risk, OR is 1.716, *p*-value < 0.001, Figure 7G) and TCGA cohort (High-risk vs. Low-risk, OR is 2.207, p-value < 0.001, Figure 7H) based on the TIDE algorithm and indicated that low-risk patients were more likely to benefit from immunotherapy. In addition to this, significantly high SUMOylation scores are observed in patients with recurrent lung adenocarcinoma (Figure 7I), and significantly high SUMOylation scores are observed in smokers (Figure 7J), suggesting that SUMOylation scores may be effective in comprehensively assessing clinical prognostic factors in patients with lung adenocarcinoma.

### 3.7 Prediction of chemotherapy drug sensitivity and construction of nomogram for lung adenocarcinoma

**FIGURE 8:**
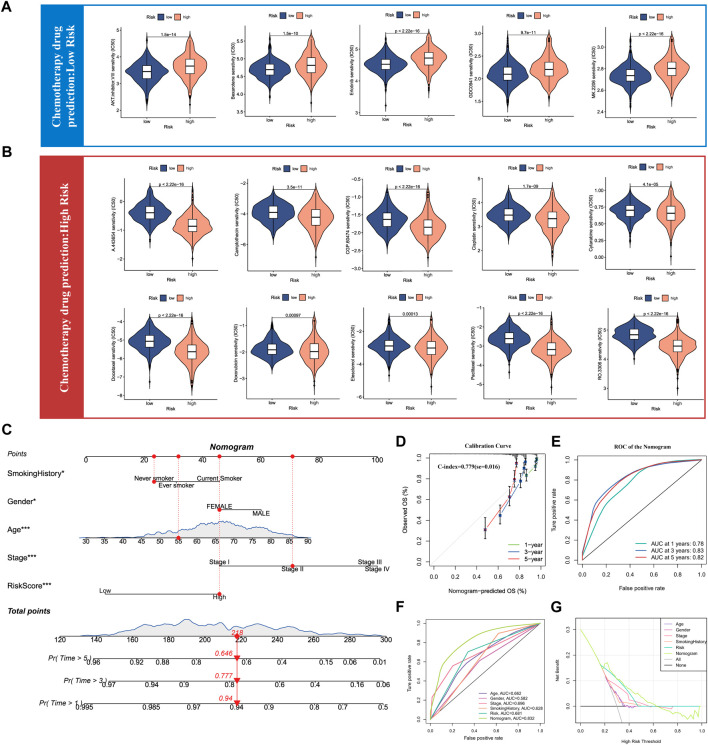
Drug sensitivity analysis of chemotherapy for lung adenocarcinoma and Nomogram construction. **(A)** Chemotherapy drug sensitivity analysis in patients with low SUMOylation score. **(B)** Chemotherapy drug sensitivity analysis in patients with high SUMOylation score. **(C)** Construction of Nomogram by combining independent prognostic features such as smoking history, gender, age, pathological stage, and SUMOylation score. **(D)** Calibration curves for evaluating the predictive performance of Nomogram for 1, 3, and 5-year OS in lung adenocarcinoma patients. **(E)** The AUC for assessing the accuracy of Nomogram to forecast 1-, 3-, and 5- years overall survival. **(F)** The AUC for predicting 3-year overall survival for comparing the accuracy of various prognostic characteristics. **(G)** Decision Curve Analysis (DCA) shows the efficacy of Nomogram and other prognostic features for clinical applications.

As the above results have confirmed that SUMOylation leads to remarkable changes in the tumor microenvironment in lung adenocarcinoma, therefore, we used the SUMOylation score to characterize the sensitivity of lung adenocarcinoma patients to various chemotherapeutic agents to guide the combination chemotherapy. Based on the mRNA levels in lung adenocarcinoma tissues, we calculated the IC50 of various chemotherapeutic agents for lung adenocarcinoma patients in different SUMOylation score groups using the pRRophetic package and found that the group with low SUMOylation score was more sensitive to AKT-inhibitor-VIII, bexarotene, erlotinib, GDC0941, MK-2206, among others than that with high SUMOylation score (Figure 8A). In contrast, the high SUMOylation score group was sensitive to more chemotherapeutic agents, including A-443654, camptothecin, CGP-60474, cisplatin, cytarabine, docetaxel, doxorubicin, elesclomol, paclitaxel, and RO-3306 (Figure 8B). These findings suggest that SUMOylation scores can accurately reflect significant biological differences between SUMOylation patterns and also correlate with clinical prognostic features.

Meanwhile, as we previously illustrated by multivariate Cox analysis that age, gender, pathological stage, smoking history, and SUMOylation score all can serve as independent prognostic factors for lung adenocarcinoma, we combined the above significant prognostic factors to construct column plots to more accurately and efficiently predict the overall survival (OS) of individual patients with lung adenocarcinoma (Figure 8C). Calibration curves showed that the OS at 1, 3, and 5 years predicted by the nomogram closely corresponded to the actual OS of lung adenocarcinoma patients (Figure 8D) and the time-dependent ROC showed high AUC of 0.78, 0.83, and 0.82 for 1-, 3-, and 5- years, respectively. Yang et al. reported that 0.9≥ AUC>0.8 indicates excellent discrimination (Shengping and Gilbert, 2017), indicating that the developed nomogram has a remarkably prognostic performance (Figure 8E). Furthermore, a comparison of the predictive performance of multiple clinical prognostic features revealed that the nomogram offered the best predictive utility relative to any other clinical feature as determined by the ROC curve (Figure 8F) as well as the DCA decision curve (Figure 8G). Finally, to further validate the accuracy of the multifactorial regression model Nomogram, we evaluated the AUC of the ROC curves of Nomogram using three independent lung adenocarcinoma cohorts and the 5-year AUC values were 0.79, 0.81, and 0.89, respectively (Supplementary Figures S7F–H7). Validation in three independent lung adenocarcinoma cohorts supports that the accuracy of our Nomogram is significantly better than a large number of reported prognostic models for lung adenocarcinoma (Mo et al., 2020; Song et al., 2020; Chen et al., 2021).

## 4 Discussion

SUMOylation performs a critical role in various biological processes encompassing immune regulation (K et al., 2021). Due to the extremely complex TME and immune background of lung adenocarcinoma, the modulatory role of SUMOylation on the TME of lung adenocarcinoma, especially on immune infiltration, is still poorly understood. Current studies are limited to individual SUMOylation regulatory genes, and the regulation of the TME mediated by integrated SUMOylation regulatory genes has not been investigated. Therefore, the identification of different SUMOylation patterns in the TME is valuable in further understanding the effect of SUMOylation on tumor immune responses in lung adenocarcinoma.

Previous studies have shown that regulation of the TME has a critical role in tumor progression and immunotherapeutic efficacy (Goliwas et al., 2021). In this study, we confirmed the tight correlation of SUMOylation regulatory genes and identified two distinct SUMOylation patterns associated with significant differences in the TME in terms of differential activation of oncogenic pathways and immune infiltration. Park S et al. classified Non-small cell lung cancer (NSCLC) into three different immunophenotypes based on tumor-infiltrating lymphocytes and immune checkpoint treatment response. Assessment of immune phenotypes is useful as a guide for determining prognosis as well as immunotherapy (Park et al., 2022). The SUMOylation patterns we identified also corresponded to distinct immunophenotypes. SUMO-C1 corresponded to a tumor immunoinflammatory phenotype with better overall survival, significant activation of immune pathways such as the IL2, inflammatory, complement, and allograft rejection pathway, and increased infiltration levels of anti-tumor immune cells. IL2 promotes not only the proliferation of cytotoxic T lymphocytes (CTLs) and natural killer cells but also the differentiation of CTLs to effector T cells (Borrelli et al., 2018). Meanwhile, CD8+T cells, Tem, cytotoxic cells, and Th17 cells were reported to promote anti-tumor immune processes (Deng et al., 2018). SUMO-C2 was consistent with reduced levels of immune infiltration and a microenvironment that promotes immune escape, corresponding to an immune-desert phenotype. Furthermore, a remarkable activation of the PI3K-AKT-mTOR signaling pathway, the cell cycle, EMT substrates, and oncogenic pathways suggested that SUMO-C2 also exhibited features of an immune exclusion phenotype. Notably, the levels of infiltration of Th2 cells, Treg cells, CD8^+^ T cells, and Th17 cells varied with different SUMOylation patterns and SUMOylation scores. SUMOylation regulatory genes are diverse in their regulation of immune cells, and the regulatory mechanism of SUMOylation regulatory genes on lung adenocarcinoma immune cells needs to be further explored. Therefore, the identification of SUMOylation patterns could help determine immunotherapy response and patient prognoses.

Notably, SUMO-C2 showed significant activation of PI3K-AKT-mTOR pathway in TME, and excessive activation of the PI3K-AKT-mTOR pathway would lead to a combined phenotype of immunodeficiency and immune dysregulation (Nunes-Santos et al., 2019), which is critical for maintaining the immunosuppressive function of Tregs and (Myeloid-derived suppressor cells)MDSCs, while inhibition of the PI3K-AKT-mTOR pathway can reduce the expression of immunosuppressive factors as well as immune checkpoint ligands (O'Donnell et al., 2018). Furthermore, we found that SUMO-C2 was associated with the increased activation of the EMT pathway and increased expression of multiple immune checkpoint molecules such as PD1. Jiang Y et al. showed that the overactivation of EMT was associated with the activation of different immune checkpoint molecules and induced tumor immune escape (Jiang and Zhan, 2020). Importantly, targeted inhibition of the PI3K-AKT-mTOR pathway maintains the antitumor immune function of CD8^+^ T cells (Patton et al., 2006), while targeted inhibition of EMT pathways has been shown to remodel the TME and restore the antitumor microenvironment (Erin et al., 2020; Kumagai et al., 2020), which suggests that targeted inhibition of the above pathways combined with immunotherapy may be a more reasonable treatment option for patients with the SUMO-C2 SUMOylation pattern.

Further, we predicted potential target drugs for high SUMOylation scores. AKT inhibitors such as AKT-inhibitor-VIII, MK-2206 (O'Donnell et al., 2018), A-443654 (Han et al., 2007), and PI3K inhibitors such as GDC-0941 (Haagensen et al., 2012) can inhibit the PI3K-AKT-mTOR signaling pathway, thereby inhibiting tumor progression. Meanwhile, CGP-60474 (Han et al., 2018), RO-3306 (Kojima et al., 2009), and doxorubicin (Jin et al., 2020) inhibit the cell cycle and DNA replication to suppress tumor progression. However, the relationship between these drugs and SUMOylation and their role in lung adenocarcinoma progression is not yet known. In addition, camptothecin (Sanchez-Alcazar et al., 2003), bexarotene (Lowe and Plosker, 2000), cisplatin (Ghosh, 2019), and paclitaxel (Wang et al., 2000) can also exert apoptosis-inducing antitumor effects, and docetaxel (Borghaei et al., 2021) has been widely used in combination with nivolumab in the treatment of NSCLC. This suggests that the SUMOylation model and score can better guide chemotherapy as well as immunotherapy for lung adenocarcinoma.

Based on two SUMOylation patterns that were associated with different TMEs, due to the complexity of SUMOylation pattern SUMO-C2 immunophenotype, we mined SUMOylation pattern-related genes (SPAGs) and further determined the genomic subtype gene-T1 corresponding to immune exclusion phenotype, which would further define SUMOylation patterns as well as TME immune phenotypes. A m6A methylation modification score can accurately determine colorectal cancer TME and immune transcripts. High m6A scores are tightly associated with worse prognosis, inferior levels of antitumor immune infiltration, and poorer response to immunotherapy (Chong et al., 2021). To determine the TME status and more precisely guide the treatment of individual patients, we present a highly sensitive prognostic model based on SUMOylation scoring, which can serve as a prognostic biomarker for lung adenocarcinoma. Noteworthy, a large number of scoring systems have been previously reported for prognostic assessment of lung adenocarcinoma, Unlike the scoring systems constructed with the DNA repair-related prognostic signature (Chang and Lai, 2019), the cell cycle-related prognostic signature (Chen et al., 2021), immune-related signature (Song et al., 2020), and the angiogenesis-related signature (Qing et al., 2022). Our model has relatively superior predictive performance compared to other signature models and can be further used as a complement to clinical factors. Interestingly, SUMOylation scores were significantly negatively correlated with immune infiltration. The immunoinflammatory phenotype consistent with SUMO-C1 exhibited a lower SUMOylation score, while the immune-desert phenotype consistent with SUMO-C2 or gene-T3 showed the highest SUMOylation score. This implies that SUMOylation can modulate the TME as well as the immune background of lung adenocarcinoma, thus regulating the progression and prognosis of lung adenocarcinoma patients.

In summary, our study identified two different SUMOylation patterns mapping different immune phenotypes and TMEs, establishing that SUMOylation can regulate the lung adenocarcinoma TME and the infiltration of immune cells. The SUMOylation score we constructed can facilitate a more accurate assessment of the TME in lung adenocarcinoma, and the nomogram based on it has good clinical utility. As samples with high SUMOylation scores showed stronger immunosuppression and immune escape, as well as promoted the activation of pro-cancer pathways, compared with low scores, we further explored two SUMOylation patterns of potential targeting agents to guide chemical combination therapy. However, our study is limited in that we relied on a few immunotherapy response cohorts with NSCLC to confirm our findings, hence, our findings must be validated in larger cohorts. Furthermore, the clinical utility of the SUMOylation score and the constructed nomogram needs to be validated in clinical settings. In addition, the characterization of the underlying mechanisms by which SUMOylation regulates the tumor microenvironment and immune background in lung adenocarcinoma and other tumors warrants further research.

## 5 Conclusion

The SUMOylation patterns can well dictate the tumor microenvironment features, particularly immune cell infiltration status, in lung adenocarcinoma. The SUMOylaiton score is indicative of the relationship between SUMOylation and immune cell crosstalk and has remarkable prognostic value, and can be used to predict immunotherapy and chemotherapy response in lung adenocarcinoma. In conclusion, the SUMOylation model and score have a high value for determining the tumor microenvironment status and prognosis prediction.

## Data Availability

The datasets presented in this study can be found in online repositories. The names of the repository/repositories and accession number(s) can be found below: https://www.ncbi.nlm.nih.gov/geo/, GSE31210 https://www.ncbi.nlm.nih.gov/geo/, GSE37745 https://www.ncbi.nlm.nih.gov/geo/, GSE50081 https://www.ncbi.nlm.nih.gov/geo/, GSE72094.

## Supplementary material

SUPPLEMENTARY FIGURE S1 Mutual association and functional annotation of 33 SUMOylation regulatory genes. GO functional annotation **(A-B)** and KEGG functional annotation **(C)** of SUMOylarion regulatory genes. **(D)** Analysis of the correlation between SUMOylation regulatory genes in lung adenocarcinoma. **(E)** Principal component analysis of SUMOylation regulatory gene expression profiles based on 57 paired lung adenocarcinoma and normal samples. **(F)** Co-mutation between regulatory genes of SUMOylation. **(G)** Multivariate cox analysis for prognostic significance among SUMOylation regulatory genes.

SUPPLEMENTARY FIGURE S2 Unsupervised clustering based on SUMOylation regulatory genes and differences in biological pathways between clusters. **(A)** Consensus clustering graph for K = 3 to 6. **(B)** The heatmap shows the mRNA expression levels of SUMOylation regulatory genes in two SUMOylation patterns. **(C)** Comparison of HALLMARK biological pathways between two SUMOylation patterns.

SUPPLEMENTARY FIGURE S3 Relationship between SUMOylation regulatory genes with their patterns and immune microenvironment. Immune cell infiltration levels between SUMOylation patterns were assessed by **(A)** EPIC algorithm and **(B)**TIMER algorithm. **(C)** Correlation between SUMOylation regulatory genes and immune infiltrating cells.

SUPPLEMENTARY FIGURE S4 GO functional annotation and key genes analysis. Functional annotation of 906 SUMOylation-associated differential genes GO, **(A)** BP, **(B)** CC, **(C)** MF. SUMOylation-related differential genes are regarded as SUMOylation Pattern-Related Genes (SPAGs). **(D)** Computation of key genes in SPAGs by Cytohubba (Degree≥5). **(E)** Consensus clustering graph with K = 2,4–9. **(F)**Comparison of significant differences in SUMOylation regulatory gene expression among three SUMOylation genomic subtypes.

SUPPLEMENTARY FIGURE S5 Construction and validation of SUMOylation score. LASSO regression analysis of 61 significantly prognostically relevant key genes , **(A,B)** partial Likelihood Deviance = 13.77, SE = 0.1393, Lambda = 0.01725. Validation of the accuracy of SUMOylation scores to predict OS at 1-, 3-, and 5-year in the independent datasets GSE31210 **(C)**, GSE37745 **(D)**, GSE50081 **(E)**, GSE72094 **(F)**, and the external validation set TCGA-LUAD **(G)**, respectively. **(H)** Multivariate cox analysis based on prognostic characteristics such as SUMOylation score in the TCGA-LUAD cohort.

SUPPLEMENTARY FIGURE S6 Validation for model genes. mRNA expression differential analysis, survival analysis, and quantitative Real-time PCR for ADRB2, **(A)** CBX7 **(B)**, CCNB2 **(C)**, CCNE1 **(D)**, CENPL **(E)**, ORC1 **(F)**, NDC80 **(G)**, and CENPN **(H)**. (**p* < 0.05, ***p* < 0.01).

SUPPLEMENTARY FIGURE S7 Relationship between SUMOylation score and immune microenvironment with other prognostic signatures. **(A)** Comparison of risk scores among genomic subtypes of SUMOylation. **(B)** Correlation of SUMOylation score with immune score and tumor purity. **(C)** Comparison of 24 immune cell GSVA scores between high- and low-SUMOylation scores. Comparison of co-stimulatory molecules **(D)** and MHC molecules **(E)** expression levels between high- and low-SUMOylation scores. Survival analysis and ROC of multivariate regression model Nomogram in independent lung adenocarcinoma cohorts with GSE31210**(F)**, GSE50081 **(G)**, GSE72094 **(H)**, the 5-year AUCs were 0.79, 0.77, and 0.89, respectively.
