# Supplementary figures and images for: SUMOylation patterns and signature characterize the tumor microenvironment and predict prognosis in lung adenocarcinoma
Source: Front Cell Dev Biol. 2023 Apr 13;11:1094588. doi: 10.3389/fcell.2023.1094588 (PMC10133499; doi:10.3389/fcell.2023.1094588)

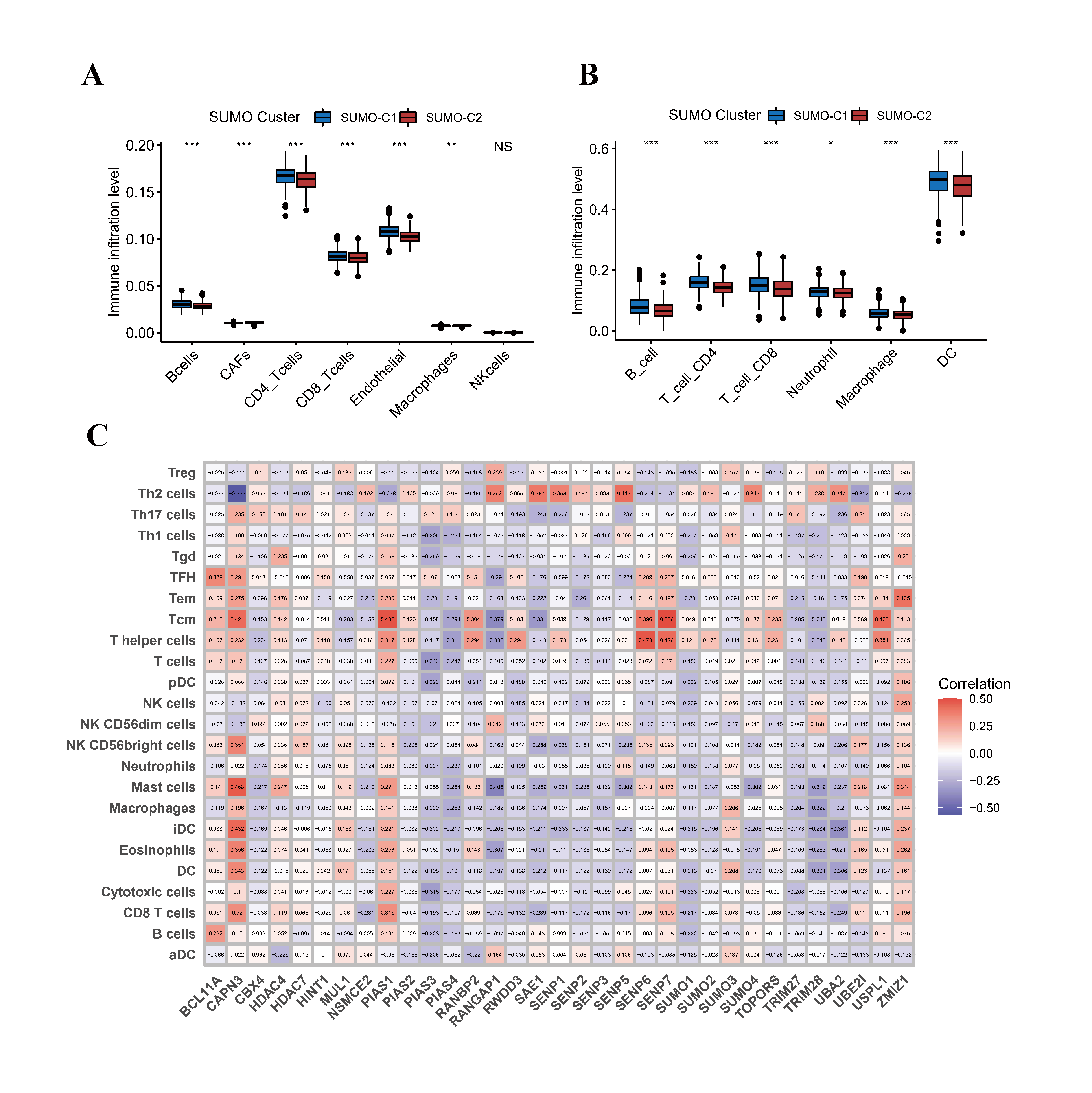

Supplement: Supplementary file 1 [file Image3.TIFF]

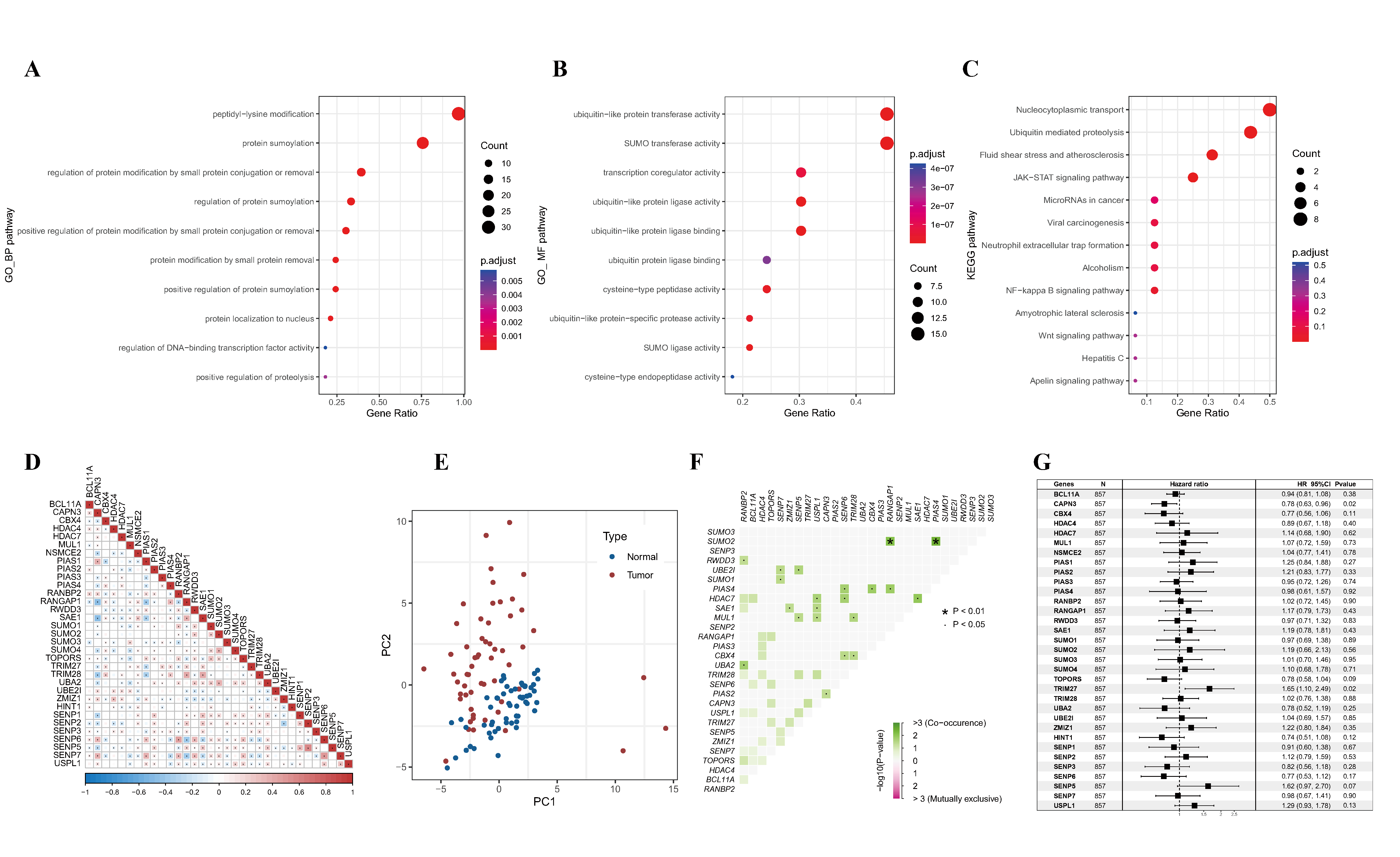

Supplement: Supplementary file 2 [file Image1.TIFF]

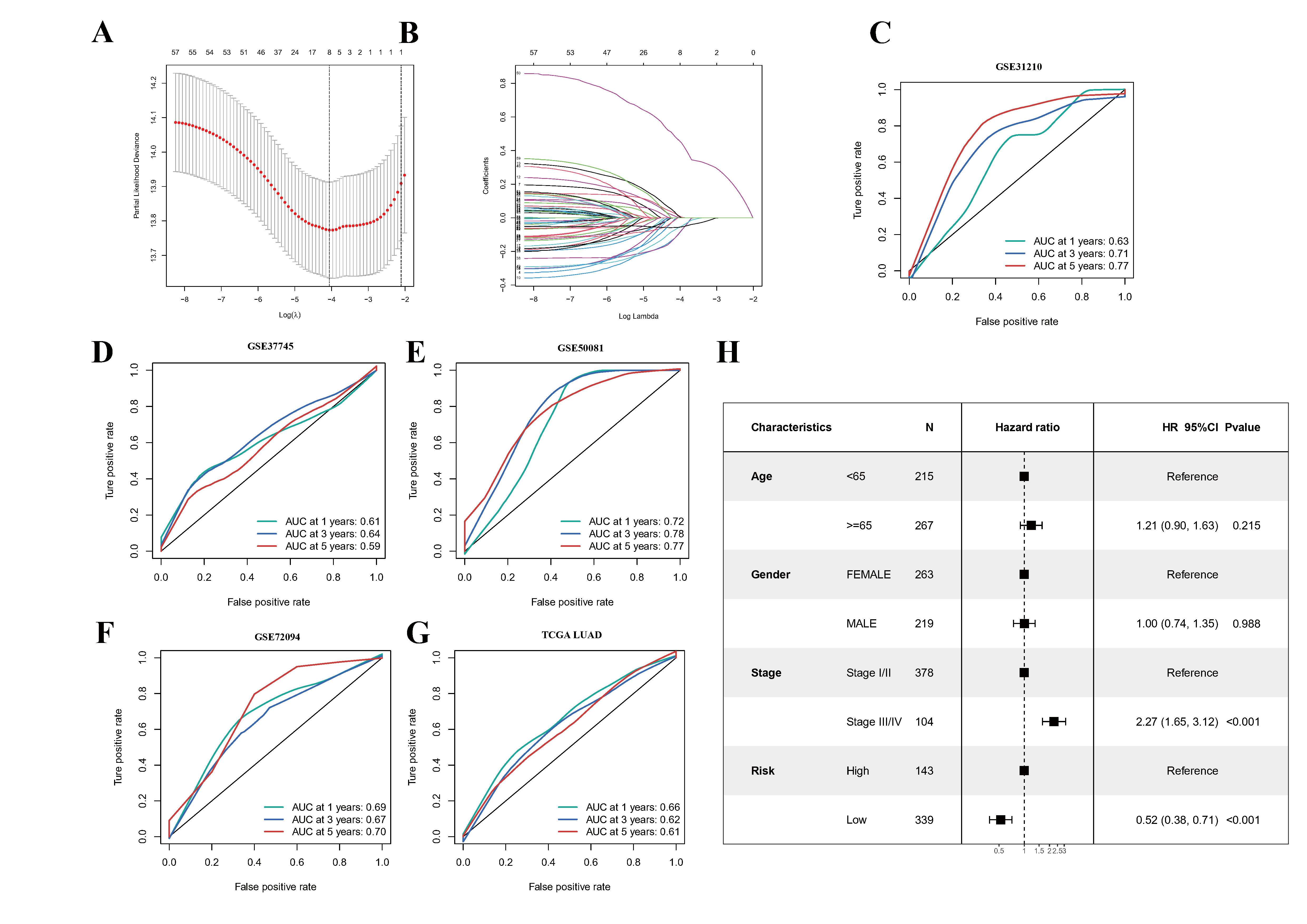

Supplement: Supplementary file 3 [file Image5.TIFF]

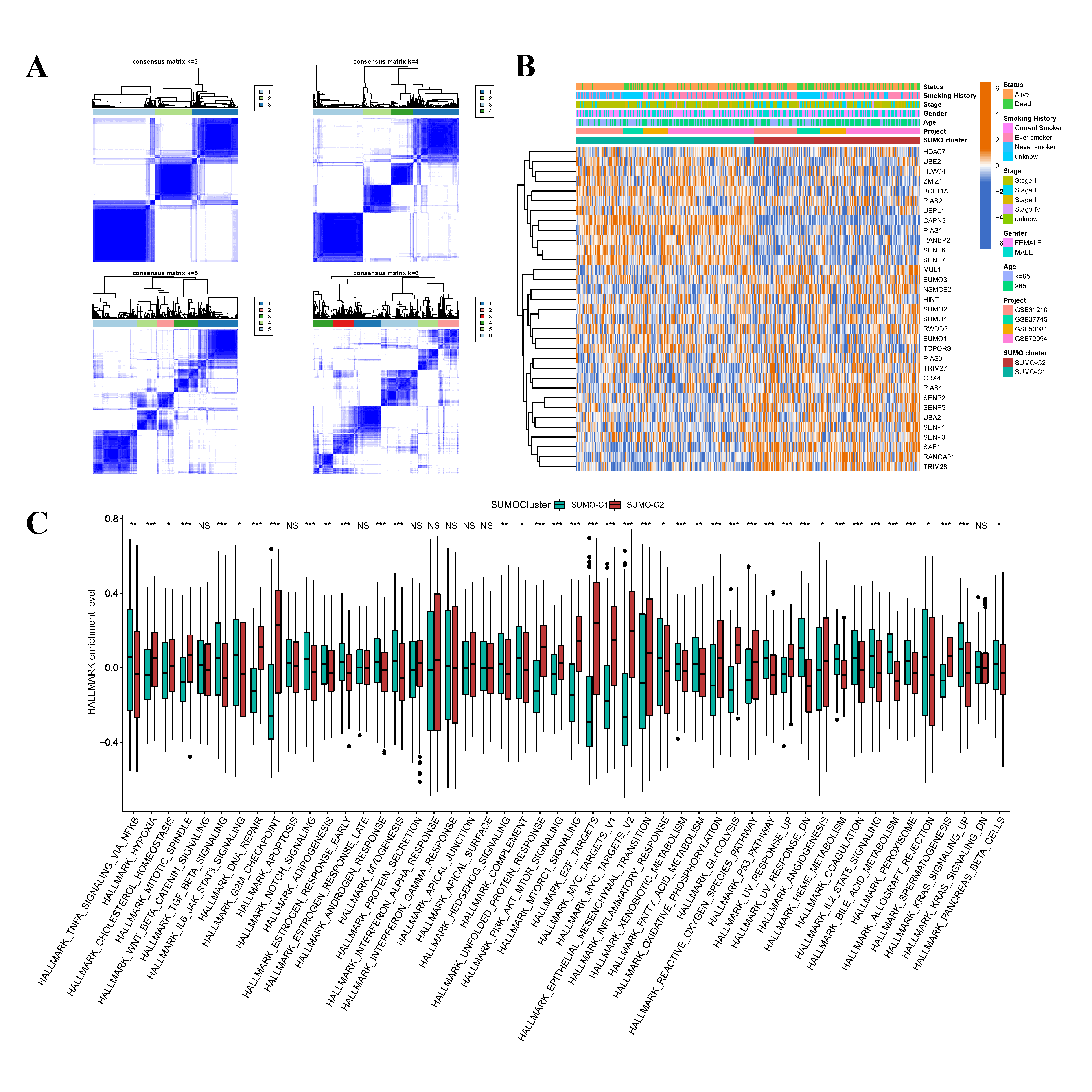

Supplement: Supplementary file 5 [file Image2.TIFF]

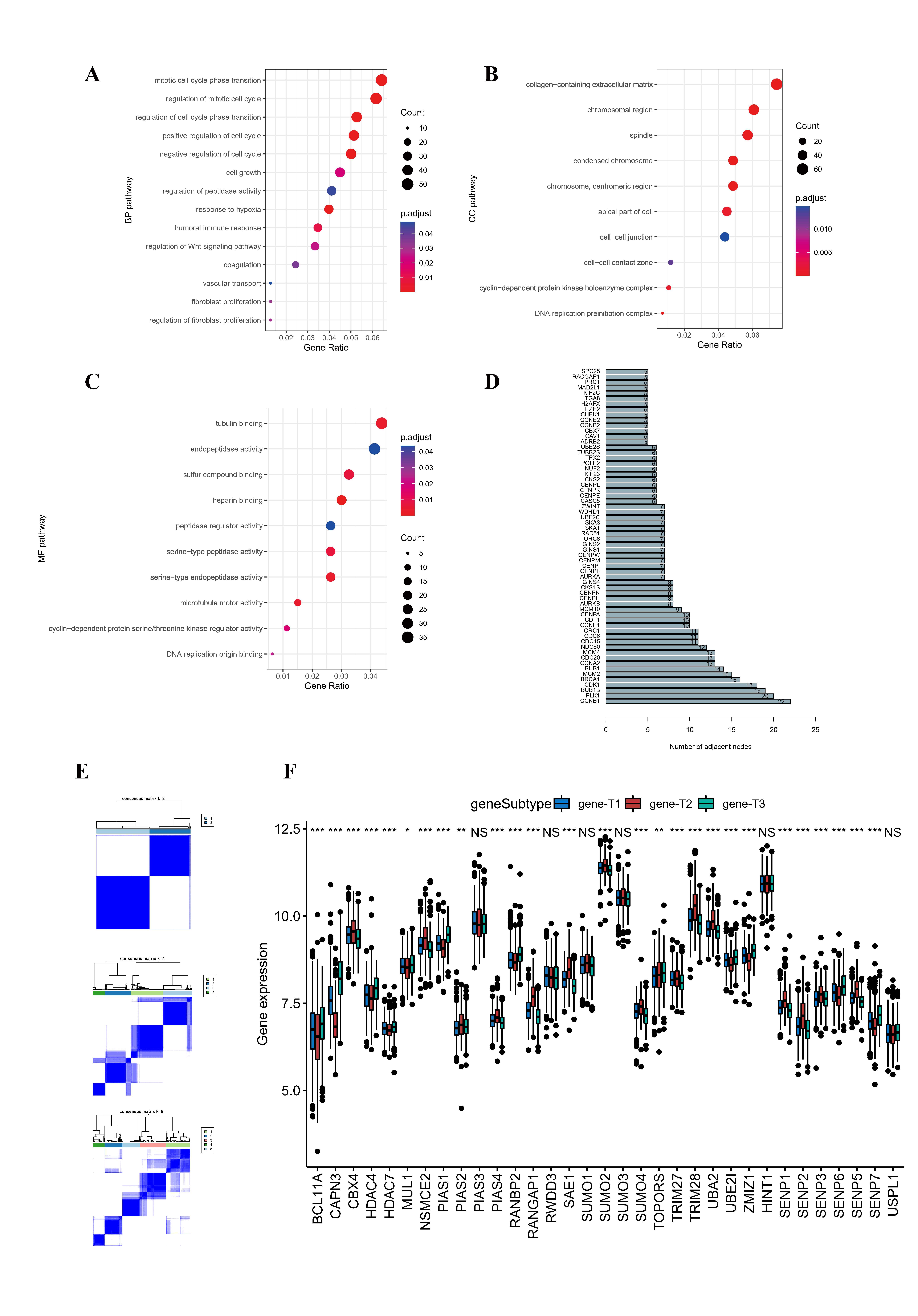

Supplement: Supplementary file 6 [file Image4.TIFF]

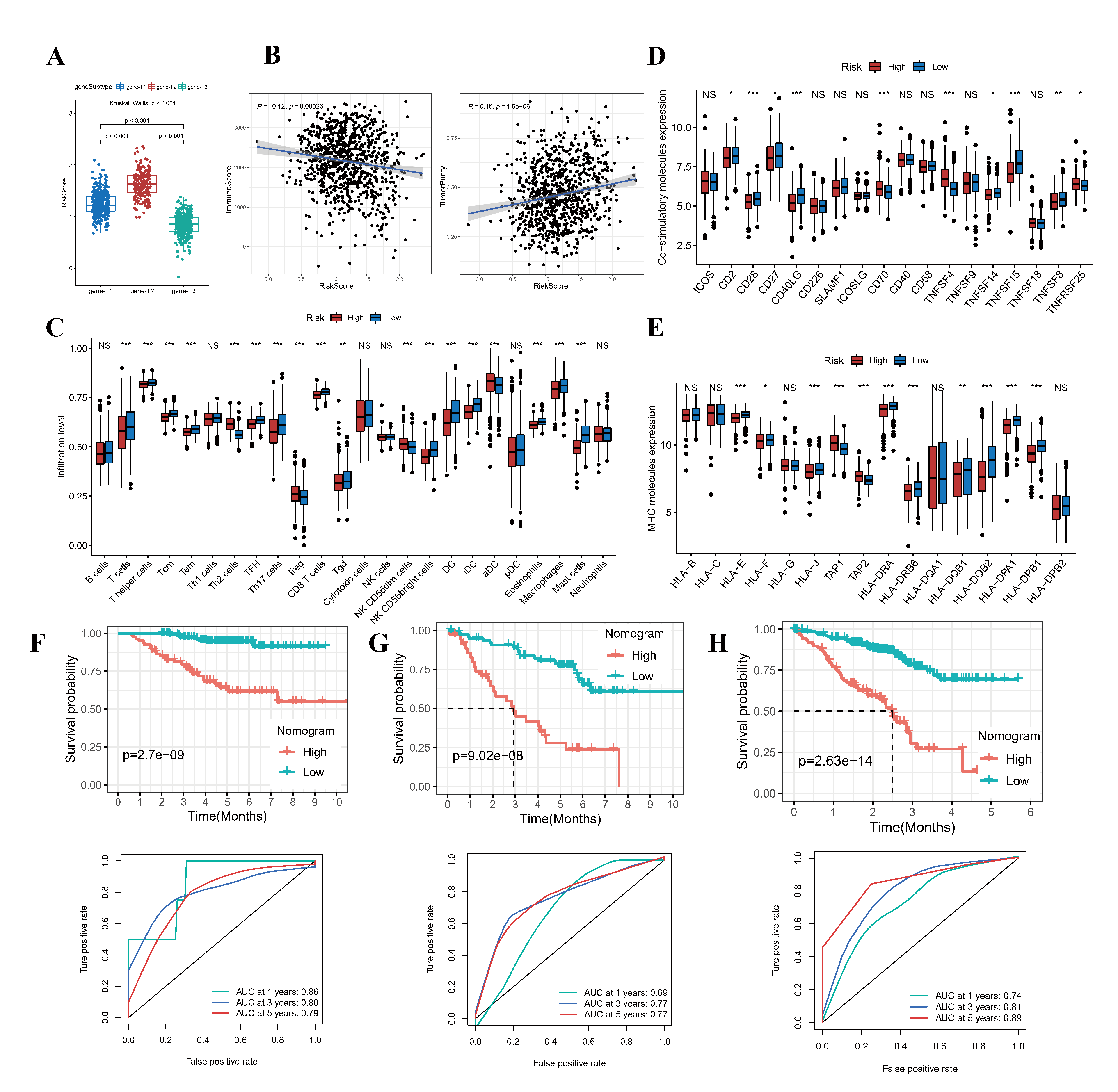

Supplement: Supplementary file 7 [file Image7.TIFF]
